# Predictors and process moderators of EMDR therapy for depressive symptoms: protocol for a series of observational N-of-1 trials

**DOI:** 10.3389/fpsyg.2025.1688526

**Published:** 2026-01-06

**Authors:** Francesca Cotardo, Emanuele Koumantakis, Pierre Gilbert Rossini, Francesca Malandrone, Paola Berchialla, Luca Ostacoli, Sara Carletto

**Affiliations:** 1 Department of Clinical and Biological Sciences, University of Turin, Turin, Italy; 2 Department of Psychology, University of Turin, Turin, Italy; 3 Psychology Unit, A.S.L. TO5, Turin, Italy; 4 Clinical Psychology Unit, A.O.U. Città della Salute e della Scienza, Turin, Italy

**Keywords:** depressive symptoms, eye movement desensitization and reprocessing (EMDR), moderators, predictors, psychotherapy, study protocol

## Abstract

**Background:**

Eye Movement Desensitization and Reprocessing (EMDR) therapy is an evidence-based intervention for trauma-related disorders and other psychological conditions, including depression. Although the positive effects of EMDR in treating depressive symptoms have been empirically supported under controlled conditions, little is known about individual and process-related factors associated with treatment outcome for depression.

**Objective:**

This study aims to present a protocol for evaluating factors associated with EMDR therapy effectiveness for depressive symptoms in real-world clinical settings. The primary objective is to identify individual characteristics that may predict treatment response. Secondly, we aim to examine potential moderating factors of EMDR for depressive symptoms, including psychologically associated symptoms and therapeutic relationship.

**Methods:**

A prospective observational design that combines a series of N-of-1 trials will be conducted in thirty-two patients presenting with clinically significant levels of anxiety, depressive, dissociative, or post-traumatic stress symptoms in at least one domain. Italian EMDR therapists voluntarily participate in the study and enroll one patient each who meets the inclusion criteria. Data will be collected via REDCap at different timepoints, from baseline to the end of therapy. A Bayesian multilevel random-effects meta-analysis and meta-regression will be conducted. This design allows for analysis of model parameters to generate direct probabilistic statements about scientific hypotheses, extending beyond treatment efficacy.

**Conclusion:**

Results will provide insights into factors influencing the effectiveness of EMDR therapy for depressive symptoms. The understanding of predictors and process moderators is expected to improve clinical interventions and contribute to bridging the current gap between research and clinical practice. Findings are expected to contribute to a promising line of research aimed to advance clinical practice through the systematic collection of real-world data. Moreover, the results may strengthen the evidence on EMDR therapy in real-world settings, and support the optimization of treatment based on individual patient characteristics.

**Clinical trial registration:**

ClinicalTrials.gov, identifier NCT07033741.

## 1 Introduction

Depression is one of the most common mental disorders, affecting approximately 280 million people worldwide (WHO, 2023).

Clinical guidelines for the management of depression emphasize that effective psychotherapy should be considered a first-line treatment for patients with mild to moderate depressive disorders (Gautam et al., 2017), as well as for chronic forms of major depression and a history of childhood trauma (Nemeroff et al., 2003).

Eye Movement Desensitization and Reprocessing (EMDR) is an evidence-based therapeutic approach developed by Francine Shapiro in 1989 to address psychological symptoms associated with traumatic memories (Shapiro, 2007; de Jongh et al., 2024).

EMDR is recognized as a cost-effective treatment for post-traumatic stress disorder (PTSD) (Chen et al., 2014; Lewis et al., 2020; Mavranezouli et al., 2020; Bisson and Olff, 2021), and strongly recommended by international guidelines for PTSD (WHO, 2013; NICE, 2018; Bisson et al., 2019; ISTSS, 2020; VA/DOD, 2023). EMDR has also shown promising outcomes when applied to various pathological conditions different than PTSD (Valiente-Gómez et al., 2017; Scelles and Bulnes, 2021). Indeed, several studies indicated that EMDR therapy effectively reduces clinically significant depressive symptoms with effects maintained at least at 3–6 months (Carletto et al., 2021; Dominguez et al., 2021; Sepehry et al., 2021; Yan et al., 2021).

Despite this evidence, little is known about the variables influencing EMDR treatment response. More broadly, there is a lack of studies exploring mediators and moderators of psychotherapy outcomes for depression. Recently, Tanguay-Sela et al. (2022) synthesized 74 patient-level predictors of response to psychological treatments for depression, including sociodemographic variables, symptom severity, comorbidity, cognitive and emotional factors, social support, and biological markers. Among the studies included in their systematic meta-review, the systematic review by Chen et al. (2019), underlined the potential of personality features, such as interpersonal functioning, attachment style, and maladaptive cognitive patterns, to inform treatment personalization and enhance treatment response. Furthermore, personality functioning tends to be described as informative risk factor for the onset, severity, and course of depression (Huber et al., 2017; Marczyk et al., 2025) and it may affect both the severity of depressive symptoms and individual responsiveness to psychotherapy (Bucher et al., 2019).

Moreover, pathogenic beliefs—trauma-related negative views of self, others, and the world—are closely linked to depression and personality disorders (Jintanachote et al., 2024). According to Beck’s cognitive theory, these beliefs increase vulnerability to depression, especially following stress (Beck et al., 1979).

Nonetheless, the EMDR framework emphasizes that addressing individuals’ belief systems is essential for treating trauma-related disorders, including depression (Hase et al., 2015, 2017). Stressful memories contribute to development and persistence of negative beliefs, which can affect treatment adherence and compromise the therapeutic relationship—a key element in the psychotherapeutic process and in reducing depressive symptoms (Yucens et al., 2014).

Indeed, Dennhag et al. (2015) found that traits like negative affectivity and alexithymia were linked to weaker working alliances, while hedonic capacity was linked to stronger ones. Similarly, Cloitre et al. (2018) demonstrated that the therapeutic relationship is a critical factor in the success of trauma-focused therapy. Hase and Brisch (2022) also emphasized its central role in EMDR therapy.

However, although research highlighted the importance of understanding personality characteristics as key factors that might predict treatment outcomes (Mulder, 2002; Dennhag et al., 2015; Samuel et al., 2018), it is still underexplored in the field of EMDR. In the present study, we conceptualize psychological disposition in terms of personality domains from the alternative model of personality proposed by the DSM-5 (APA, 2013), along with pathogenic personal beliefs as cognitive patterns of internalized adverse experiences that may influence therapeutic process (Aafjes-van Doorn et al., 2021).

Furthermore, despite the positive effects of EMDR in treating distress-related disorders have been empirically supported under well-controlled conditions (Chen et al., 2014; Yan et al., 2021), research on mediators, moderators, and predictors associated with EMDR outcomes in real-world clinical practice remains an evident challenge and is worth exploring to enhance knowledge and guide treatment decisions (Matthijssen et al., 2020; Ramallo-Machín et al., 2024).

To fill this gap, there is a need of real-world studies to better understand the therapeutic process, identifying factors influencing the efficacy of EMDR therapy for depressive symptoms, and capturing individual variability in treatment response.

N-of-1 trials are well adapted to this context, where individual variability in treatment response is substantial and personalization is critical. In this design, each patient serves as their own control, allowing for the generation of rigorous, individualized evidence on treatment effects. Moreover, aggregating a series of N-of-1 trials yields estimates of treatment effect at population level. This is well suited to reduced clinical samples, as it increases statistical power through repeated within-subject observations.

### 1.1 Study objectives

#### 1.1.1 Primary objectives

The primary objective of this study is to identify potential predictors of EMDR therapy outcomes in individuals with depressive symptoms in real-world clinical settings. More specifically, we hypothesized that a lower individual psychological functioning at baseline, with a high dysfunctional personality domains and pathogenic personal beliefs, influence the effect of EMDR in treating depressive symptoms. Moreover, we aim to assess whether changes in individual psychological functioning during therapy modify therapy outcomes.

#### 1.1.2 Secondary objectives

The secondary objective is to examine other potential moderators of the EMDR for depressive symptoms. We hypothesized that psychological associated symptoms (anxiety, dissociation, post-traumatic symptoms, emotional dysregulation, and sleep difficulties) at baseline are related to a smaller reduction in depressive symptoms. Furthermore, since the therapeutic relationship is described as a core element of EMDR therapy, we intend to evaluate the role of working alliance in treatment progress and outcomes.

## 2 Methods and analysis

### 2.1 Study design

We will conduct a prospective observational study through an adaptation of the N-of-1 trial design, which consists of conducting a multiple crossover clinical trial within a single patient, including randomized periods of treatment and control with repeated outcome measurements to determine individual treatment effects (Guyatt et al., 1990). In particular, we will aim to reflect real-world clinical practice, observing the natural evolution of EMDR treatment patterns in each of our enrolled patients. Following the framework outlined by Vock et al. (2024), a detailed follow-up protocol will be implemented to assess treatment outcomes, document clinically relevant events during and between sessions, and identify key factors which potentially influence the effectiveness of EMDR therapy, including both positive responses and cases of non-response, at strategically selected time points.

To comprehensively track both process- and outcome-related variables over time, multiple data collection timepoints will be employed, including baseline, pre-session, and post-session assessments, as well as evaluations at predefined follow-up intervals.

**Figure 1:**
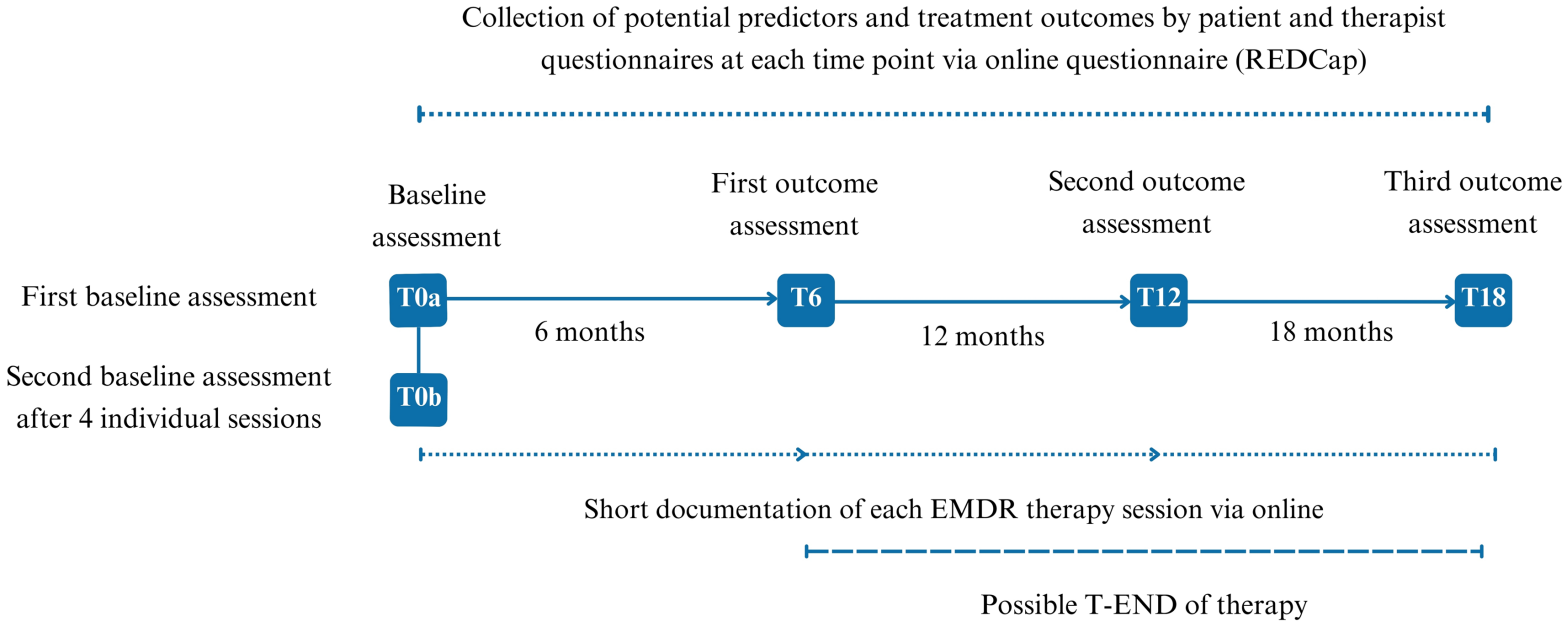
Flow chart of data acquisition. T0a, Baseline assessment; T0b, Second baseline after four individual sessions; T6-T12, 6 months outcomes assessments; T-END, final assessment, occurring at any time between T6 and maximum at T18, based on therapy duration.

The data acquisition steps are outlined in Figure 1.

Standardization will be promoted through clinician training workshops, adherence to a unified EMDR therapy protocol, and regular feedback sessions to ensure data consistency among therapists and patients.

We will report results according to the CONSORT Extension for N-of-1 Trials (Vohra et al., 2016), adapted for the observational, non-randomized design. Items related to randomization, allocation concealment, or predefined treatment sequences will be marked as not applicable. To ensure transparent reporting of observational aspects, the CENT checklist will be used in combination with STROBE (Vohra et al., 2016; von Elm et al., 2008).

This study has been approved by the Ethical Committee of the University of Torino (approval number 0565145) and it has been registered at ClinicalTrials.gov (ID: NCT07033741).

### 2.2 Participants

The study sample will include both EMDR therapists and their patients, with specific inclusion criteria designed to ensure methodological rigor and the reliability of outcomes.

#### 2.2.1 Therapists

Eligible therapists are certified members of the EMDR Italian National Association to ensure adherence to the established standards of practice of EMDR therapy and consistency in the delivery of therapy. Therapists will identify patients who meet the study inclusion criteria from their routine clinical practice and facilitate enrollment.

Each therapist will identify a single patient (1:1 ratio) during the initial phase of psychological consultation. Therapists will enroll the first eligible patient with whom a new course of psychotherapy is initiated and who meets predefined inclusion criteria, is considered appropriate for EMDR therapy, and agrees to participate in the study. Once identified, therapists will guide patients in providing informed online consent via a protected link to the Research Electronic Data Capture (REDCap) (Harris et al., 2009).

#### 2.2.2 Inclusion/exclusion criteria

Inclusion criteria are (a) being 18 years of age or older, (b) who give informed consent, and (c) presenting clinically significant symptoms in at least one of the following domains: anxiety, depression, dissociative, or post-traumatic stress symptoms.

Individuals presenting with severe neurological impairment or other severe psychiatric conditions will be excluded.

### 2.3 Data collection

Data will be collected and managed using REDCap, ensuring that both the therapist and patient are officially enrolled and ready to contribute data. REDCap is based on a robust data security model. It provides an intuitive user interface and support for the rapid development and deployment of electronic data capture tools. All information stored in REDCap will be treated with personal identifiers replaced by unique study codes to protect the privacy of the therapists. The project configuration decides that the responses of patients do not require them to identify themselves, granting them full anonymity.

Sociodemographic and clinical information are collected prior to the clinical assessment. Patient data include age, gender, marital and educational status, occupational situation, living arrangement, previous and current psychotherapeutic treatment, and medication use; therapist data include educational background, clinical experience, theoretical orientation, EMDR training level, and supervision activities.

The assessment of clinically significant symptoms will be conducted using validated screening tools according to the objectives of the study. A full baseline evaluation will be performed at the initial session (T0a), where patients will complete a set of baseline self-report questionnaires evaluating personality traits, symptomatology, physical and emotional functioning, and other factors that could be relevant to the study objectives (detailed in Table 1). A second baseline assessment (T0b) will be conducted after the completion of four therapy sessions, focusing on emotional regulation, personal beliefs, attachment style, and traumatic memories processing. Both the patient and therapist will evaluate the therapeutic alliance at T0b, offering insights into the evolving dynamics of the therapeutic process.

**Table 1:** Measures applied at different points of data acquisition.

Instruments	Time for completion													
	T0a		T0b		Pre-session^1^		Post-session^2^		T6		T12		T-END	
	P^3^	T^4^	P	T	P	T	P	T	P	T	P	T	P	T
Basic sociodemographic data	X	X												
BDI-II	X								X		X		X	
PHQ-9	X		X		X				X		X		X	
PID-5-BF	X												X	
PBS-R			X										X	
GAD-7	X		X		X				X		X		X	
DES-II			X						X		X		X	
MDI			X						X		X		X	
PITQ			X	X					X	X	X	X	X	X
PCL-5			X						X		X		X	
DERS			X						X		X		X	
MSQ	X								X		X		X	
WAI			X	X					X	X	X	X	X	X
LSC-R	X													
CEAS	X								X		X		X	
PHLMS	X								X		X		X	
RQ			X										X	
PDS^5^										X		X		X
Recalled Memory Evaluation^6^							X		X		X		X	
Resource and Event Assessment^7^								X						

1 Pre-session: before each session.

2 Post-session: at the end of each session.

3 P: Patient.

4 T: Therapist.

5 Completed by the therapist after the first four EMDR sessions and at T6, T12, and T-END.

6 Completed by the patient before and after EMDR processing sessions targeting one of their top 10 traumatic memories (selected during assessment, EMDR phase 3) or newly identified core negative events (EMDR phases from 1 to 3).

7 Completed by the therapist after each therapy session.

BDI-II, Beck Depression Inventory; PHQ-9, Patient Health Questionnaire-9; PID-5-BF, Personality Inventory for DSM-5 – Brief Form; PBS-R, Pathogenic Beliefs Scale-Revised; GAD-7, Generalized Anxiety Disorder-7; DES-II, Dissociative Experiences Scale; MDI, Multidimensional Dissociation Inventory; PITQ, Progress in Treatment Questionnaire; PCL-5, Post-traumatic Stress Disorder Checklist; DERS, Difficulties in Emotion Regulation Scale; MSQ, Mini Sleep Questionnaire; WAI, Working Alliance Inventory; LSC-R, Life Stressor Checklist-Revised; CEAS, Compassionate Engagement and Action Scales; PHLMS, Philadelphia Mindfulness Scale; RQ, Relationships Questionnaire; PDS, Processing Difficulties Scale.

Following baseline assessments (T0a and T0b), additional assessments at 6 months (T6), 12 months (T12), with a maximum follow-up period of 18 months (T18) will be used to monitor changes in symptomatology, physical and emotional functioning, and other potential predictive factors. If therapy concludes before T18, the final assessment will be conducted at that time point as a comprehensive post-treatment follow-up to ensure all outcome measures are captured.

From T0b onward, pre-session and post-session data collection will capture detailed longitudinal records of therapy progress. Before each therapy session, patients will complete questionnaires assessing anxiety and depression symptoms and tracking changes in traumatic memories on account of treatment. After each session, therapists will document session-specific data, including process-related factors and specific features of the EMDR session, such as the therapeutic target, the technique of bilateral stimulation used, and the subjective units of distress. Potential adverse events in the patient’s life (e.g., self-harm and suicidality) between treatment sessions are also recorded.

### 2.4 Measurements

The Beck Depression Inventory (BDI-II, Beck and Steer, 1993) is a 21-item self-report instrument used to assess the severity of depressive symptoms based on DSM-IV criteria. The total score ranges from 0 to 63, with higher scores indicating greater levels of depression. A score above 13 is considered the cut-off for depressive symptoms (14–19: mild depression; 20–28: moderate depression; ≥29: severe depression).

The Patient Health Questionnaire-9 (PHQ-9, Kroenke et al., 2001) is a 9-item self-report questionnaire to measure depressive symptoms in the past 2 weeks based on DSM-IV and DSM-5 major depressive disorder. Participants rate the frequency of symptoms on a scale from 0 (“not at all”) to 3 (“nearly every day”), with total scores indicating no symptoms (0–4 points), mild symptoms (5–9 points), moderate symptoms (10–14 points), moderately severe (15–19 points), severe symptoms (≥20 points). A score ≥ 10 is typically considered indicative of clinically significant depressive symptoms. The Personality Inventory for DSM-5 – Brief Form (PID-5-BF, Krueger et al., 2013) is a 25-item self-report measure to assess five personality domains: negative affect, detachment, antagonism, disinhibition, and psychoticism. Each domain consists of 5 items rated on a 4-point scale ranging from 0 (“very false or often false”) to 3 (“very true or often true”). The total score ranges from 0 to 75, with higher scores indicating greater personality dysfunction, while individual domain scores range from 0 to 15. Consistently high scores in a particular domain may indicate significant and challenging areas for the patient that may warrant further assessment, treatment, or follow-up.

The Pathogenic Beliefs Scale-Revised (PBS-R, McCollum et al., 2024) is a 21-item self-report measure to evaluate the presence of a maladaptive personal beliefs and the distress associated with them on a 4-point Likert scale, ranging from 0 (“not at all”) to 3 (“highly”). The PBS-scale provides four scores: total score, undeserving, cannot rely on others, and guilt.

The Generalized Anxiety Disorder-7 (GAD-7, Spitzer et al., 2006) is a 7-item self-report instrument designed to measure the frequency of generalized anxiety symptoms over the previous 2 weeks on a scale from 0 (“not at all”) to 3 (“nearly every day”). Total scores indicate no symptoms (0–5 points), mild symptoms (6–10 points), moderate symptoms (11–15 points), or severe symptoms (greater than 15 points).

The Dissociative Experiences Scale (DES-II, Carlson et al., 1993) is a 28-item, self-report assessment tool designed to measure dissociative experiences, including emotional involvement, depersonalization, derealization, compartmentalization, and amnesia. Each item on the scale evaluates the proportion of time an individual experiences the specific symptom. The overall score is calculated as the average of all item scores, ranging from 0% (“never”) to 100% (“always”).

The Multidimensional Dissociation Inventory (MDI, Briere et al., 2005) is a 30-item self-report tool designed to assess dissociative phenomena across six domains: Disengagement, Depersonalization/Derealization, Emotional Constriction, Memory Disturbance, and Identity Dissociation. Each item is rated on a 5-point Likert scale ranging from 0 (“never”) to 4 (“very often”).

The Progress in Treatment Questionnaire – Patient (PITQ-p, Schielke et al., 2017) is a 32-item self-report measure completed by patients, focusing on their ability to manage emotions, symptoms, relationships, safety, and overall well-being. Patients report the percentage of time they exhibit specific behaviors during the previous week, using a scale ranging from 0% (“never”) to 100% (“always”). Items 27–32 are specifically designed for patients who experience dissociative self-states. Higher scores indicate better adaptive functioning. The PITQ-p enables patients to reflect on their progress and use of coping strategies while providing therapists with valuable insights to guide treatment planning and evaluate treatment responsiveness.

The Progress in Treatment Questionnaire – Therapist (PITQ-t, Schielke et al., 2017) is a 29-item measure designed to assess the percentage of time patients demonstrate treatment target behaviors over the prior 6 months. Each item is rated on a scale ranging from 0% (“never”) to 100% (“always”). The questionnaire includes six specific items (items 24–29) for patients exhibiting dissociative self-states. Higher scores reflect greater adaptive functioning. The PITQ-t helps therapists evaluate multiple areas of patient functioning, providing a structured approach to monitoring progress and refining treatment plans.

The Post-traumatic Stress Disorder Checklist (PCL-5, Blevins et al., 2015) is a 20-item self-report assessment designed to evaluate the presence and severity of PTSD symptoms. Each item is rated on a 5-point Likert scale, where 0 indicates the absence of symptoms and 4 indicates extreme severity. The items of the PCL-5 are designed to be aligned with the DSM-5 diagnostic criteria for PTSD. The total score ranges from 0 to 80, with higher scores indicating greater levels of PTSD symptoms. A cutoff score of 33 is considered a reasonable threshold for provisional PTSD diagnosis.

The Difficulties in Emotion Regulation Scale (DERS, Gratz and Roemer, 2004) is a 36-item, self-report questionnaire to assess how individuals perceive and manage relevant difficulties in their emotional experiences. It is related to various aspects of emotion regulation, including acceptance of emotional responses, impulse control, emotional clarity, and the use of emotion regulation strategies. Respondents are asked to rate their agreement on a 5-point Likert scale ranging from 1 (“strongly disagree”) to 5 (“strongly agree”).

The Mini Sleep Questionnaire (MSQ, Natale et al., 2014), is a 10-item self-report tool for screening sleep disorders. Each item is rated on a 7-point Likert scale, ranging from 1 (“never”) to 7 (“always”). Four levels of sleep quality are identified: 10–24 (good sleep quality), 25–27 (mild sleep difficulties), 28–30 (moderate sleep difficulties), and ≥ 31 (severe sleep difficulties).

The Working Alliance Inventory (WAI, Horvath and Greenberg, 1989) is a 36-item self-report measure to assess the quality of the therapeutic alliance available in both therapist (WAI-t) and patient (WAI-p) versions. The scale evaluates three fundamental dimensions of the therapeutic relationship: goals (agreement on therapy objectives), tasks (agreement on the steps or methods to achieve these goals), and bond (the emotional connection and mutual trust between therapist and patient). Each item is rated on a 7-point Likert scale ranging from 1 (“never”) to 7 (“always”), with higher scores reflecting a stronger working alliance. Subscale scores provide detailed insights into the specific components of the alliance, while the total score represents the overall strength of the therapeutic relationship.

To gain better insight into potential moderators and predictors of the effectiveness of the EMDR therapy process, participants will also complete the following self-report measures.

The Life Stressor Checklist-Revised (LSC-R, Wolfe et al., 2012) will be used. LSC-R is a self-report measure used to evaluate the cumulative effects of life stressors, such as natural disasters, interpersonal violence, and the sudden death of a loved one. A dichotomous response format (yes/no) is used to indicate whether individuals have experienced each event. For endorsed events, patients provide additional details, including the age of occurrence, whether they felt in harm’s way or helpless (“yes” or “no”), and rate the event’s impact and associated emotional distress on a 5-point Likert scale (1 = “not at all” to 5 = “extremely”). Individuals are also asked to identify up to three events that currently have the greatest impact on their lives.

The Compassionate Engagement and Action Scales (CEAS, Gilbert et al., 2017) is a self-report measure designed to assess suffering and commitment to alleviate and prevent it. The CEAS comprises three subscales: compassion for others, compassion from others, and self-compassion. Each subscale contains thirteen items, divided into two components: engagement (e.g., noticing, being emotionally moved, tolerating distress) and action (e.g., taking steps to help, expressing care, seeking solutions). Responses are rated on a 10-point Likert scale, ranging from 1 (“never”) to 5 (“always”). Higher scores reflect greater levels of sensitivity and commitment within each dimension of compassion.

The Philadelphia Mindfulness Scale (PHLMS-I, Cardaciotto et al., 2008) is a 20-item self-report inventory designed to measure two core components of mindfulness: awareness (attentiveness to present-moment experiences) and acceptance (a non-judgmental attitude toward thoughts and emotions). Each subscale comprises 10 items rated on a 5-point Likert scale ranging from 1 (“never”) to 5 (“very often”), reflecting the frequency of the subjective experiences. Subscale scores are calculated as the sum of their respective items, with higher scores indicating greater mindfulness abilities.

The Relationships Questionnaire (RQ, Bartholomew and Horowitz, 1991) is a 4-item self-report tool used to assess an individual’s adult attachment style. Each item describes one of four prototypical attachment patterns (secure, fearful, preoccupied, and dismissing) and individuals will rate their agreement or disagreement with four statements on a 7-point Likert scale ranging from 1 (“strongly disagree”) to 7 (“strongly agree”). The RQ identifies whether an individual has a secure, anxious, or avoidant attachment style and provides insights into how they approach and experience interpersonal relationships.

To evaluate therapy sessions and to track patient progress, therapists will complete the following questionnaires.

The Processing Difficulties Scale (PDS, Ramallo-Machín et al., 2024) is a 17-item self-report instrument designed to evaluate challenges encountered during memory processing in EMDR therapy, following the standard protocol. Clinicians rate each item on a 5-point Likert scale ranging from 0 (“never”) to 4 (“always”), assessing specific processing styles. These styles include indicators of poor processing characterized by a lack of generalization, effective general processing, unproductive emotional processing, and signs of loss of dual attention during sessions. The PDS provides valuable insights into the quality and style of processing, aiding clinicians in identifying potential barriers to therapeutic progress.

The two tools below were designed for this study to systematically capture clinical information typically collected during psychotherapy and standard EMDR practice. The Recalled Memory Evaluation is a 14-item self-report instrument designed to assess the emotional, cognitive, and sensory aspects of memory recall after EMDR sessions (e.g., clarity, vividness, sounds, and smells). Patients rate their experience on an 11-point Likert scale ranging from 0 (“not at all”) to 10 (“completely”). The form provides key insights into changes in memory processing over time, helping to share and deepen understanding of patients’ lived experiences of EMDR.

The Resource and Event Assessment is a therapist-completed measure designed to document significant clinical events and session characteristics after each therapy session. It evaluates interim events since the previous session (e.g., self-harm, stressful or positive life events, and resource utilization) and identifies the focus of the current session (e.g., stabilization, resource development, or target processing). Responses are recorded using dichotomous options (“yes” or “no”).

### 2.5 Treatment

Participants will receive EMDR therapy in accordance with standardized clinical protocol (Hofmann et al., 2015; Shapiro, 2018). In line with the real-world nature of this study, the number of EMDR sessions will not be *a priori* defined. However, after each session, therapists will specify the session type (EMDR or non-EMDR) in REDCap. Therapists will exercise clinical judgment in selecting session type, and the exact session date, type, and key contextual variables (e.g., symptom severity, medication changes, major life events) will be recorded to permit adjustment for time-varying confounding in the analysis.

### 2.6 Sample size estimation

There is no established method for estimating sample size on N-of-1 trial designs in observational settings. Following Senn’s (2019) recommendations, and the practical guidance of Yang et al. (2021), at least 32 therapist-patient dyads, with a minimum of 26 visits per participants, approximately 1 visit/week for 6 months. Yang et al. suggests that roughly 24 repeated measurements per individual provide reasonable power to detect a moderate within-patient effect (Cohen’s d around 0.5), when aggregating results across multiple N-of-1 series. We expect a small number of patients to record fewer than 24 outcome measurements. However, since follow-up can extend up to 18 months, some patients will likely record considerably more measurements. Moreover, because the Bayesian hierarchical model naturally down-weights individual patients with less precise within-patient estimates, participants with fewer observations will contribute less to the pooled effect. As a result, a small number of shorter series should not materially reduce the precision of the overall estimate.

### 2.7 Statistical analysis

We will begin with a descriptive analysis of demographic, clinical, and outcome variables. Quantitative variables will be summarized using the mean and standard deviation if the assumption of normality is met; otherwise, the median and interquartile range will be reported. Qualitative variables will be described using absolute counts and percentages.

Differences between personality and personal beliefs groups will be evaluated using appropriate statistical tests: Student’s t-test for normally distributed quantitative variables, Mann–Whitney U test for non-normally distributed quantitative variables, and either the Chi-square test or Fisher’s exact test for categorical variables, as appropriate.

Sessions with missing values on treatment (i.e., EMDR or no-EMDR session) or outcome (i.e., PHQ-9 final score) will be excluded from the following analyses.

To assess the impact of psychological functioning at baseline on the base of personality domains and pathogenic personal beliefs on EMDR’s effect on depressive symptoms, we will combine data from multiple N-of-1 trials using a Bayesian multilevel time-series model (Schmid and Yang, 2022).

Session-level outcomes (PHQ-9 scores) are nested within patients. The model will include patient-specific random intercepts and random slopes for the EMDR effect. To model temporal dependence, residuals at the session level will incorporate an AR(1) structure, capturing correlation between consecutive measurements within each patient. Weakly informative priors will be used for all regression coefficients (Normal[0,5]) and for the standard deviations of random effects (half-Normal[0,2]), while the AR(1) correlation parameter will have a uniform prior on (−1,1). Missing session-level outcomes will be handled directly within the hierarchical framework under a missing-at-random assumption, allowing all available repeated measurements to contribute to estimation. Patients with fewer sessions will be naturally down-weighted via partial pooling, without the need for imputation. Sensitivity analyses will explore alternative residual correlation structures (e.g., ARMA[1,1]), inclusion/exclusion of patients with very few sessions, and adjustment for time-varying covariates to assess potential confounding. Model convergence will be assessed through trace plots, $\hat{R}$statistics ($\hat{R}$≤1.01), and effective sample size for all parameters. Posterior predictive checks will be used to evaluate model fit and the adequacy of assumptions.

We will also perform secondary analyses using anxiety symptoms as outcome, and will evaluate the effect of other predictors and moderators (e.g., dissociation, post-traumatic symptoms, emotional dysregulation, and sleep difficulties). Missing sessions will be handled directly in the longitudinal model. Because the primary analysis uses a Bayesian hierarchical time-series model, missing outcome data are accommodated under a missing-at-random (MAR) assumption without requiring imputation or exclusion of the entire individual. Dropout will be recorded by therapists together with the stated reason, and dropout patterns will be summarized descriptively.

All analyses will be conducted using R version 4.5.0 (R Core Team, 2025).

## 3 Discussion

The present study protocol is the first prospective observational design that combines multiple N-of-1 trials to investigate the predictors and process moderators of the efficacy of EMDR therapy for depressive symptoms. Previous studies have shown the importance of understanding how EMDR works when it is delivered under real-world conditions (Matthijssen et al., 2020). We intend to evaluate processing styles and their relationship with various indicators that may help improve clinical interventions (Ramallo-Machín et al., 2024) and contribute to bridging the current gap between research findings and clinical practice (Fernández-Álvarez et al., 2020).

The inclusion of patients presenting at least one or more of the clinical domains (e.g., anxiety, depression, dissociative, or post-traumatic stress symptoms) is strongly supported by evidence that these conditions are not separate entities but rather mutually influential systems that constitute an integrated network structure (Groen et al., 2020; Lazarov et al., 2020). This network is heavily defined by a general distress factor or negative affect that is common to depression, generalized anxiety disorder, and PTSD (Price et al., 2019). Depressive symptomatology is intrinsically linked to this structure. The inclusion of dissociation is also critical because it is another important condition that may influence the effectiveness of treatment in patients with anxious and depressive symptoms who are resistant to previous treatment (Prasko et al., 2016). A higher degree of dissociation at the beginning of the treatment predicted minor improvement in depression and anxiety symptom reduction, while the major therapeutic change is connected to greater reduction of the dissociation level (Fung et al., 2025; Prasko et al., 2016). Therefore, including these interacting domains is necessary to accurately explore factors associated with the EMDR treatment effect on depressive symptoms.

Through the integration of innovative methodologies using N-of-1 trials, meta-analysis and meta-regression, this study contributes to deepening our understanding of factors influencing the effectiveness of EMDR and its application in natural settings. Additionally, the design of our study offers the opportunity to explore both patient and therapist characteristics and process variables in real-world clinical settings, with a broad range of measures assessed on multiple occasions. Indeed, as Kazdin (2007) suggested, assessment on a session-by-session basis (i.e., every time before and after each session over the course of treatment) is useful for evaluating the mediator of change and symptom reduction and considering individual differences during these changes.

For our first objective, we expect that profiling individual psychological functioning at baseline will be associated with the severity of depressive symptoms at baseline and during EMDR therapy. Although several studies have examined the relationship between personality and mental health treatment (Bucher et al., 2019), the literature has shown a limitation in assessing personality dimensions only at the beginning of therapy (Samuel et al., 2018). For these reasons, we expect that collecting personality and pathogenic belief ratings at the end of therapy will have the potential advantage of capturing more data for therapists and allowing clearer prediction of longer-term treatment outcomes in clinical reality.

Moreover, the structure of our study supports real-world evidence through the longitudinal evaluation of other potential factors that potentially play an important role in adapting effectiveness of EMDR treatment. Indeed, these findings could inform a more nuanced understanding of the mediating role of individual and process factors of EMDR therapy in associations with depressive symptoms severity.

In line with the broader aim of this project, we expect that this study will represent a promising line of research that has been designed to advance clinical practice by enabling the systematic collection of real-world data and developing evidence-based strategies to optimize interventions based on individual patient characteristics. We believe that a more thorough understanding of the factors associated with the efficacy of EMDR therapy and treatment implications will enhance the evaluation of individual variability in treatment responses, offering critical insights into the factors underlying the effectiveness of EMDR therapy in depressive symptoms. Finally, through this collaborative effort between researchers and clinicians, we intend to promote and advance the quality and scientific evidence of EMDR in the treatment of depression by emphasizing the interplay between therapeutic processes and treatment outcomes within a real-world context.

## Trial registration and status

This study has been registered at Clinicaltrials.gov (ClinicalTrials.gov Identifier: NCT07033741). Data collection began in June 2025 and is currently ongoing. The study is expected to be completed by December 2027. The study timeline is presented in the Supplementary material.
